# Engineering iridoviruses: development of reverse genetics and virus rescue systems

**DOI:** 10.1128/jvi.01852-24

**Published:** 2025-04-17

**Authors:** Daria Vladimirova, Daniela Kunecova, Mariana Nascimento, Ji Yoon Kim, Dusan Kunec, Jakob Trimpert

**Affiliations:** 1 Institut für Virologie, Freie Universität Berlin 9166, Berlin, Germany; 2 Department of Diagnostic Medicine and Pathobiology, College of Veterinary Medicine, Kansas State University 5308, Manhattan, Kansas, USA; Northwestern University Feinberg School of Medicine, Chicago, Illinois, USA

**Keywords:** ranaviruses, bacterial artificial chromosome, frog virus 3, TAR cloning

## Abstract

Iridoviruses are a family of large DNA viruses that infect insects and poikilotherm vertebrates, including amphibians, reptiles, and fish. Notably, members of the genus *Ranavirus* cause mass mortality in fish and amphibians, threatening aquaculture and contributing to global amphibian decline. Despite their omnipresence and impact, key aspects of iridovirus biology remain unknown, largely due to the absence of reverse genetics systems. In this study, we developed, characterized, and utilized a reverse genetics system for frog virus 3 (FV3, *Ranavirus rana1*), one of the most widely studied iridoviruses. The rescued virus exhibits growth and phenotypic properties identical to those of the parental virus isolate. Furthermore, we established an alternative approach of virus reconstitution from genomic DNA, utilizing a heterologous iridovirus as a helper. This novel approach enables rapid and facile rescue of modified viruses from naked DNA. The reverse genetics and rescue systems described in this study will advance iridovirus research by facilitating efficient genetic modification of the virus genome in yeast or bacteria. This could clear the path to elucidating functions of virus genes and allow a much more detailed understanding of iridovirus biology. Moreover, owing to the promiscuous nature of FV3 with its ability to infect hosts from different classes of animals, the FV3 system has the potential to serve as a platform for the development of modified live vaccines for a variety of fish and amphibian species.

**IMPORTANCE:**

Iridoviruses pose a substantial threat to aquaculture and global amphibian populations, yet research has been hindered by the lack of a reverse genetics system. In this study, we describe the development of the first such system for this virus family. We constructed a synthetic clone of frog virus 3 (FV3) that can be propagated and genetically manipulated in both yeast and bacteria, yielding a virus that has biological properties identical to the parental virus isolate. Furthermore, we developed a novel helper virus-based system for the rescue of FV3 from purified DNA. This system provides an essential tool for advancing our understanding of iridovirus biology and serves as a platform for the development of modified live virus vaccines.

## INTRODUCTION

Iridoviruses are a diverse family of viruses within the phylum *Nucleocytoviricota*, comprising large double-stranded DNA viruses that often replicate in both the nucleus and cytoplasm. The family *Iridoviridae* currently contains seven genera placed within two subfamilies (1). Viruses from the *Alphairidovirinae* subfamily infect poikilotherm vertebrates such as amphibians, reptiles, and fish, while members of the *Betairidovirinae* predominantly infect invertebrates (2–4). Among the *Alphairdovirinae,* the genus *Ranavirus* is particularly known for its ecological and economic impact (5–7). Notably, ranaviruses are one of the leading causes of mass mortality events among amphibians globally (8–10), with ever-increasing outbreak zones and new species affected (11–14). Ranaviruses thus contribute to the drastic decline in amphibian populations, which has made this class of animals the unfortunate number one in rankings of extinct and endangered species (15, 16). However, substantial aspects of the biology of iridoviruses, and specifically ranaviruses remain unknown (4, 8, 17–19). Studying the biology of iridoviruses has been hampered by the lack of reverse genetics systems. In fact, to date, not a single reverse genetics system has been published for the entire class *Megaviricetes*. Consequently, researchers must rely on traditional methods based on homologous recombination in infected cells to generate recombinant viruses (17, 20). This makes generating virus mutants time-consuming and delays scientific progress in the understanding of iridovirus biology. Iridovirus research would benefit from a robust reverse genetics system that would enable easy and reliable manipulation of viral genome in bacteria.

In this study, we developed a reverse genetics system for frog virus 3 (FV3), one of the most widely studied members of the *Ranavirus* genus (2, 4, 17, 21). Our genetics system is based on a bacterial artificial chromosome-yeast artificial chromosome (BAC-YAC), assembled by transformation-associated recombination (TAR) cloning in yeast (22–26). TAR cloning is a powerful genetic technique that facilitates the selective isolation and cloning of large DNA molecules in yeast *Saccharomyces cerevisiae*. This method leverages the high efficiency of homologous recombination to capture target sequences into a YAC vector. The viral genome can be assembled from overlapping fragments (27, 28) or cloned in its entirety using a single-step approach (29).

To generate a full-length bacterial FV3 DNA clone, we divided the entire FV3 genome into 14 approximately equally-sized overlapping fragments, which we amplified from the FV3 DNA by PCR. We then assembled the fragments with the linear BAC-YAC vector, which allows selection and propagation of the assembled DNA construct in both yeast *S. cerevisiae* and bacterium *Escherichia coli*. Applying this method, we generated hundreds of independent FV3 BAC-YAC clones and selected three of them for further characterization.

Like other large DNA viruses, such as the African swine fever virus and poxviruses (30, 31), iridoviruses cannot be rescued from intact full-length viral DNA in permissive cells upon transfection (1, 32, 33). This creates a significant technical challenge, as infectious viruses must be rescued through more labor-intensive approaches. However, it is known that iridoviruses can be rescued from naked DNA with the help of a homologous UV-irradiated helper virus (34). Here, we present an alternative route of rescue that employs a replicating heterologous helper virus. This approach facilitates the rescue and decreases the likelihood of homologous recombination between the genomes of the cloned and the UV-irradiated virus during the reconstitution. Our reverse genetics system serves as a powerful tool for fast and reliable genetic manipulation of the FV3 genome, thus paving the way for advanced studies in viral pathogenesis and functional genomics. Moreover, this system also has the potential to serve as a platform for vaccine development.

## RESULTS

### Establishing an iridovirus reverse genetics system using TAR cloning

**Fig 1:**
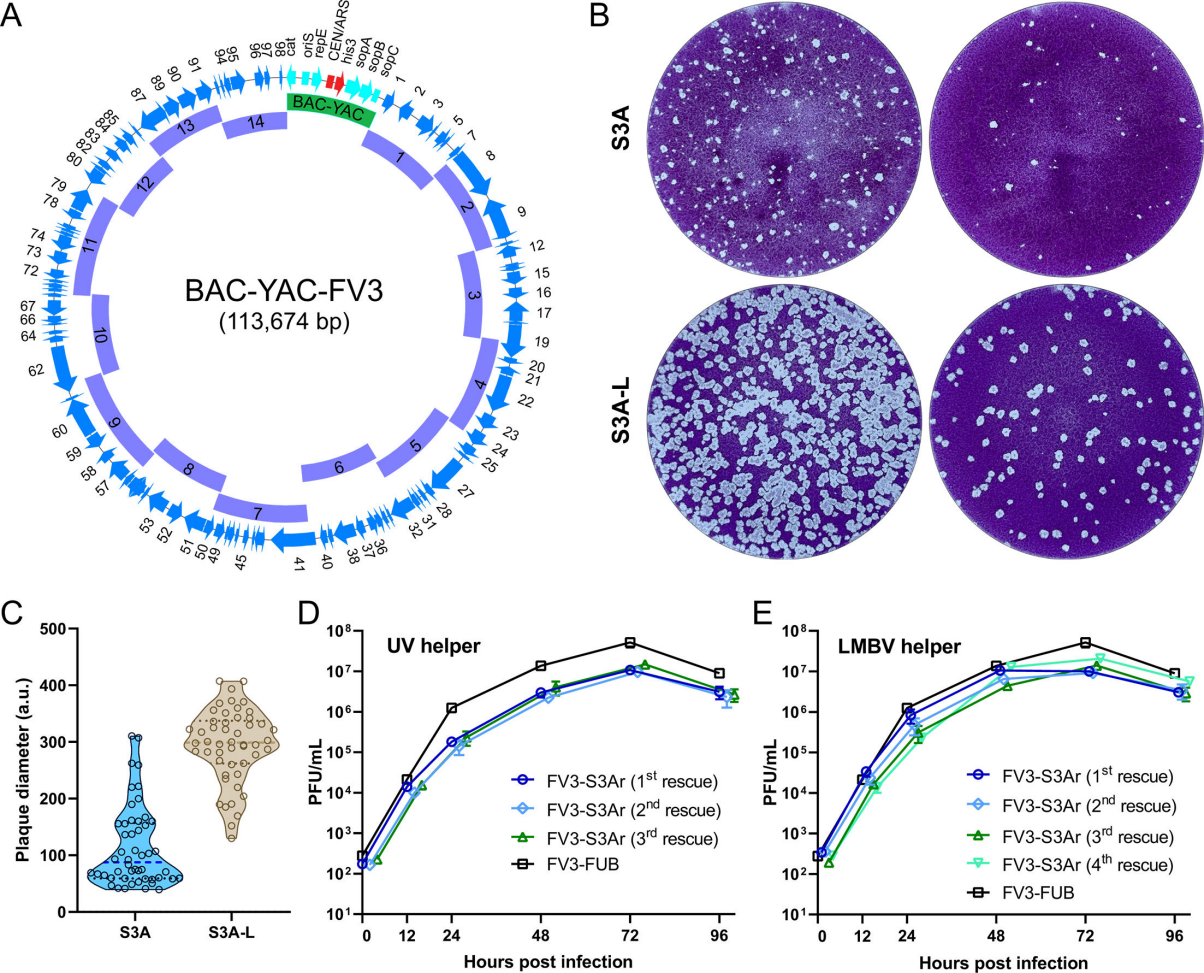
Reverse genetics system of FV3. (**A**) Graphical overview of the recombinant BAC-YAC FV3 construct, with a total length of 113,674 bp. The construct was assembled from 14 overlapping subgenomic FV3 fragments (purple bars) and the 7,771 bp BAC-YAC TAR vector (green bar). The blue arrows denote the 98 FV3 open reading frames (ORFs) (**B**) Plaques produced by FV3 rescued from the bacterial clone S3A with the UV-irradiated helper virus. The upper panel shows the plaque phenotype of “large and small” plaques from passage 3 after reconstitution (S3A), while the lower panel shows the plaque phenotype following the purification of large plaques (S3A-L). (**C**) The truncated violin plot shows diameters of 50 randomly selected plaques shown in panel B. The dashed line indicates the median, and the dotted lines indicate the first and third quartiles. (**D and E**) Multi-step growth kinetics of parental FV3 (FV3-FUB) and FV3-S3Ar. FV3-S3Ar was independently rescued three times using UV-irradiated FV3 (**D**) and four times using largemouth bass virus (LMBV) as a helper virus (**E**). Mean virus titers were determined for each time point using two biological and two technical replicates by plaque assays. Error bars indicate SD. No significant differences were detected between the growth of the rescued viruses and the parental virus by one-way analysis of variance.

To address the need for a versatile reverse genetics system in iridoviruses, we generated a full-length infectious clone of FV3 employing TAR cloning in yeast. In TAR cloning, the entire viral genome is assembled via homologous recombination between overlapping viral DNA fragments and the YAC vector (22–26). Because cloned sequences can be very efficiently manipulated using diverse and well-established tools of bacterial genetics, we opted for a dual bacterial and yeast vector, essentially a BAC-YAC hybrid (Fig. 1A). This vector enables the propagation of cloned sequences in both yeast and *E. coli*. In addition, it is possible to obtain BAC-YAC DNA of much better quality from *E. coli* compared to yeast, which is advantageous for further downstream processes, such as restriction fragment length polymorphism (RFLP) analysis, sequencing, or virus rescue.

The FV3 isolate, designated FV3-FUB, generously provided by Jacques Robert from the University of Rochester, was propagated on BHK-21 cells. We determined the sequence of this isolate by next-generation sequencing (NGS) and deposited it in the DNA Data Bank of Japan (DDBJ) under accession number LC830689. The sequence of FV3-FUB was highly similar to the FV3 GenBank reference sequence (NC_005946.1), with only minor sequence variations (Table S1). Some of these variations have been identified in other FV3 isolates, such as *Rana sylvatica* ranavirus (RSR) and a FV3-like isolate from a spotted salamander in Maine (SSME) (35, 36). For example, one of these variations is a deletion of a single nucleotide (C) in the last codon at the 3′ end of ORF50L, which results in the in-frame fusion of ORF50L with ORF49L, giving rise to ORF49/50L.

Based on this sequence, we designed primers (Table S2) to amplify the entire FV3 genome as 14 overlapping fragments (Fig. S1). The amplified fragments were approximately 8 kb long and overlapped each other by 99–287 bp. To minimize the number of unintentional mutations in amplified fragments and, thus, also in the final reverse genetics system of FV3, we amplified the fragments using a high-fidelity VeriFi polymerase (PCR Biosystems). The gel-purified fragments were assembled by TAR cloning in the *Saccharomyces cerevisiae* strain VL6-48N (22, 28). The assembly was highly efficient. From several hundred colonies obtained, we randomly selected 10 yeast colonies, isolated yeast DNA, and transformed it into *E. coli*. Next, we isolated BAC-YAC DNA, screened via RFLP assay, and selected three independently generated clones (S3A, R1A, and R2A), which appeared to contain the entire FV3 genome for further analysis (FV3 BAC-YAC, Fig. S2A and B).

Subsequently, we attempted to rescue the replicating virus from clones S3A, R1A, and R2A using UV-irradiated FV3 as a helper virus. While we successfully rescued the virus from all clones, clone S3A exhibited a phenotype most closely resembling the parental virus isolate, with a more pronounced cytopathic effect (CPE) compared to other clones. Next, we sequenced clone S3A using Illumina short-read sequencing and identified that the assembled S3A construct differed from the predicted sequence only in two single nucleotide polymorphisms (SNPs), with one silent mutation and one amino acid change affecting ORF85R (A72T, Table 1). Additionally, we subjected this clone to Oxford Nanopore-based long-read sequencing, which confirmed the short-read sequencing results and revealed the absence of one of the three 24 bp tandem repeats in the gene ORF49/50L (Table 1). We hypothesize that the deletion is unlikely to have a significant effect on the function of ORF49/50L protein, as the deletion results in an in-frame deletion of eight amino acids, but the protein still retains two other sets of eight amino acids from the remaining tandem repeats, potentially preserving its overall structure and function. A previous study reported that ORF49L encodes an immediate-early protein containing a SAP DNA-binding domain, while ORF50L encodes a delayed-early protein with unknown function (37).

**TABLE 1:** Sequence variation between the FV3 isolate FUB and the engineered FV3 BAC-YAC clones S3A, R1A, and R2A*^a^*

No.	Position (FV3 FUB)	FV3 FUB	S3A	R1A	R2A	Mutation	Location	Effect
1	55189–55212	24 bp	–**^*b*^**	24 bp	24 bp	24 bp deletion	ORF49/50L	In-frame deletion
2	65923	C	T	C	C	C→T	Intergenic	Non-coding region
3	92699	G	**A** *^c^*	G	G	G→A	ORF85R	A72T
4	7243	C	C	T	C	C→T	ORF7R	Silent
5	65436	A	A	G	A	A→G	ORF58R	Silent
6	95498	C	C	A	C	C→A	ORF87L	K107N
7	6227	A	A	A	T	A→T	ORF6R	K74M
8	45994	G	G	G	T	G→T	Intergenic	Non-coding region
9	94588	G	G	G	A	G→A	ORF87L	Silent

^*a*^ Highlighted in gray are differences between compared sequences.

^*b*^ This 24 bp sequence is absent (deleted) in clone S3A.

^*c*^ The mutation highlighted in bold and underlined indicates a mutation that was corrected by targeted mutagenesis to create clone S3Ar.

To evaluate the growth properties of FV3 rescued from clone S3A (FV3-S3A), we performed multi-step growth kinetics and observed a perceptible growth impairment of the FV3-S3A compared to the parental virus (Fig. S3A). In addition, the plaques produced by the FV3-S3A presented two discernibly different phenotypes: smaller and larger parent virus-like plaques (Fig. 1B and C). Therefore, we decided to evaluate the replication properties of viruses forming small and larger plaques through plaque purification and growth kinetics (Fig. S3B). While all viruses isolated from larger plaques consistently produced plaques in size similar to the parental FV3, we were unable to obtain a replication-competent virus from the small-sized plaques.

We surmised that the reduced replication of FV3-S3A could be caused by the amino acid mutation A72T in ORF85R. Therefore, we repaired this mutation by *en passant* mutagenesis in *E. coli* (38), resulting in the clone S3Ar. Next, we assessed the growth properties of FV3-S3Ar derived from the repaired clone FV3-S3Ar by multi-step growth kinetics (Fig. 1D and E). We found that FV3-S3Ar exhibited the growth properties of the parental FV3. Consequently, the repaired clone S3Ar was selected as the parental bacterial clone for the subsequent construction of mutants, and its sequence was deposited in the DDBJ under accession number LC830690.

The sequencing of the BAC-YAC clones R1A and R2A revealed that these clones also contained a minimal number of unintended mutations (Table 1). The clone R1A exhibited two silent SNPs and one SNP causing a single amino acid change, while the clone R2A exhibited one intergenic SNP, one silent SNP, and one SNP causing an amino acid change. These results confirm the feasibility of assembling complete iridovirus genomes through TAR cloning with only a limited number of mutations when the overlapping genomic fragments are produced using a high-fidelity polymerase. In addition, screening a larger number of clones may potentially identify those that are completely error-free.

### FV3 reporter expressing EGFP

To test our reverse genetics system and to determine whether it is feasible to rescue infectious virus mutants from the engineered DNA constructs, we generated an FV3 reporter mutant by replacing the coding sequence of gene ORF64R with the coding sequence of the enhanced green fluorescent protein (EGFP) of *Aequorea victoria*. To this end, we replaced the coding sequence of ORF64R from start to stop codon with the coding sequence of EGFP by *en passant* mutagenesis in *E. coli* (38). We chose to make this construct as proof-of-concept because the same viable EGFP reporter virus was previously engineered through homologous recombination in eukaryotic cells (20). The ORF64R encodes a putative viral caspase activation and recruitment domain-containing (CARD) protein. The CARD protein is known as a virulence factor, as an FV3 mutant lacking this gene has been shown to have delayed growth kinetics in natural host cell line, as well as an attenuated course of disease in infected *Xenopus laevis* tadpoles (20).

### Rescue of infectious FV3 from DNA using homologous UV-irradiated helper virus

**Fig 2:**
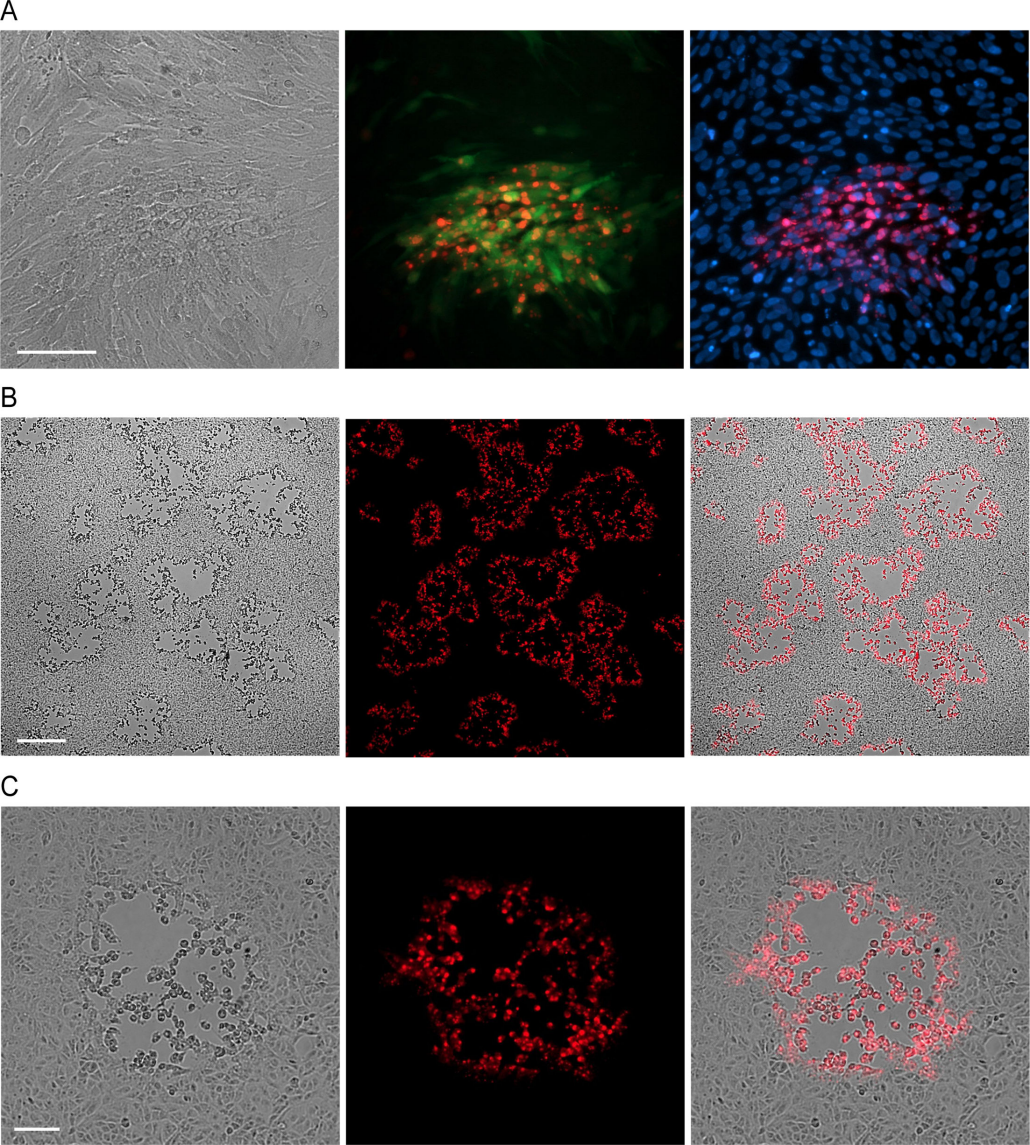
The FV3 reverse genetic system readily gives rise to infection progeny on Vero E6 and BHK-21 cells. (**A**) Foci generated by FV3-Δ64R-EGFP marker virus on BHK-21 cells 2 days after infection. The left panel shows cells under normal light, and the middle and the right panels display images under fluorescent light. Infected BHK-21 cells produce EGFP (green). The major capsid protein is visualized with the mouse monoclonal antibody (red/fuchsia), and cell nuclei are stained with DAPI (blue). Bar, 0.1 mm. (**B and C**) CPE induced by FV3 on Vero E6 cells, rescued from the S3Ar clone, facilitated by LMBV, 5 days after infection. The widespread infection of the cell monolayer (**B**) and a single FV3 plaque (**C**) are shown. Infected cells were stained with the mouse monoclonal anti-epizootic hematopoietic necrosis virus (EHNV) and goat anti-mouse IgG Alexa Fluor 647 antibodies (red). The left, middle, and right panels contain bright-field light, fluorescent, and merged images, respectively. Bar in B, 0.5 mm; bar in C, 0.1 mm.

Iridoviruses are notorious for their inability to be rescued from naked DNA transfected into permissive host cells (1, 32, 34). It is speculated that an essential but enigmatic transactivator of immediate-early transcription must be present in transfected cells to initiate virus replication (2, 32, 33). To address this limitation, we used the EGFP construct to determine if the infectious virus can be rescued from naked DNA with the help of UV-irradiated homologous helper virus, as previously described (31, 34). For this purpose, we treated BHK-21 cells with the UV-irradiated FV3-FUB and then transfected the purified bacterial FV3-Δ64R-EGFP DNA into the cells using the Lipofectamine 3000 transfection reagent. Two days after transfection, widespread EGFP fluorescence could be observed in transfected cells (Fig. 2A). CPE characteristic for FV3 developed rapidly within 3 to 4 days and was associated with EGFP expression. These results reconfirmed that it is possible to rescue infectious viral progeny of cloned DNA with the help of UV-irradiated parental virus. The obtained results also demonstrated that bacterially-derived FV3 DNA can give rise to infectious virus and that our reverse genetics system is indeed suitable for the generation of various FV3 mutants.

While we could recover FV3-Δ64R-EGFP from purified bacterial DNA with the help of UV-irradiated FV3, the rescue was only successful when the UV inactivation of the helper virus was somewhat incomplete. This observation aligns with prior research suggesting that prolonged UV exposure inactivates some factor(s) required for successful reactivation, possibly the virion-associated transcriptional transactivator (39). We observed that the UV-irradiated helper virus alone caused CPE typical of FV3 infection on BHK-21 cells, although with significant delay. Thus, following infection with UV-treated FV3, plaques became visible 2 weeks after infection, whereas cells infected with WT virus exhibited noticeable CPE within 2 to 3 days. Furthermore, we determined that FV3 subjected to longer UV exposure times neither induced CPE nor could be used as a helper virus because treatment of BHK-21 cells with this over-irradiated virus failed to induce the rescue of FV3-Δ64R-EGFP from naked DNA.

### Rescue of infectious FV3 from naked DNA using a heterologous fish ranavirus

The observation that the irradiated helper virus must be, to some degree, replication-competent to facilitate FV3 reconstitution from DNA prompted concerns regarding contamination of the rescued virus with the helper virus and potential recombination between the two viruses. This issue would be specifically problematic for viruses lacking a fluorescent marker or those that grow less efficiently than the helper virus.

Given that the irradiated helper virus retains some residual replication capacity, plaque purification of the rescued virus may still be required to obtain the desired recombinant virus without the admixture of the irradiated homologous helper virus. Therefore, we investigated an alternative method of FV3 rescue from DNA. We hypothesized that FV3 could be rescued using largemouth bass virus (LMBV, *Ranavirus micropterus1*), a heterologous fish ranavirus. LMBV, first isolated in 1995 (40, 41), shows only about 70%–78% sequence identity with FV3 and displays a marked rearrangement in gene order (2, 7, 21, 41–44).

To distinguish between the two viruses in cell culture, we first confirmed that FV3-infected cells could be visualized by immunofluorescence (IF) staining using a murine monoclonal antibody raised against epizootic hematopoietic necrosis virus (EHNV), a closely related ranavirus. To validate the specificity of the IF staining, we confirmed that this antibody cross-reacts only with FV3 (Fig. 2B and C) and does not recognize LMBV, as visible plaques produced by LMBV on BF-2 cells remained unstained.

**Fig 3:**
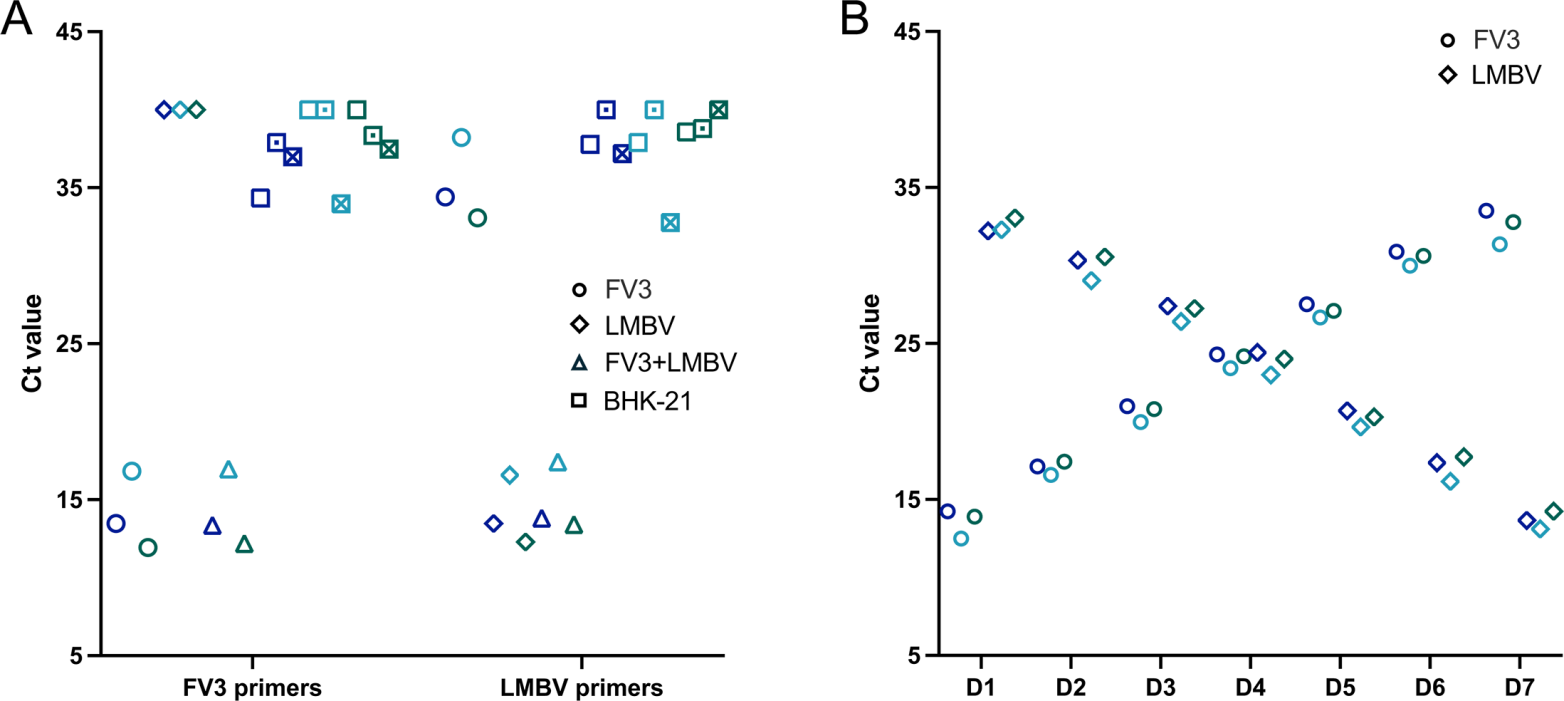
Validation of qPCR assays for the detection of FV3 and LMBV genome sequences. (**A and B**) qPCR assays were performed on biological triplicates and run in technical duplicates. Replicate 1 is shown in dark blue, replicate 2 in turquoise, and replicate 3 in green. The data are plotted as the means of technical duplicates. The graphs for all three biological replicates and their corresponding technical duplicates are presented in Fig. S4A and B. (**A**) The specificity of the qPCR assays for discriminating FV3 (circles) and LMBV (diamonds) was evaluated using samples containing only FV3 or LMBV DNA, as well as samples containing DNA from both viruses (triangles). Negative controls containing DNA from uninfected BHK-21 cells were run in nine replicates (represented by squares, squares with dots, and crossed squares). (**B**) The cross-reactivity of the qPCR assays for FV3 (circles) and LMBV (diamonds) was assessed using samples containing varying ratios of both viral genomes. The tested dilutions were D1 (FV3:LMBV = 1,000 ng:0.001 ng), D2 (100 ng:0.01 ng), D3 (10 ng:0.1 ng), D4 (1 ng:1 ng), D5 (0.1 ng:10 ng), D6 (0.01 ng:100 ng), and D7 (0.001 ng:1,000 ng). The linearity (R²) of the pooled biological replicates, calculated using R (45), was 0.987 for FV3 and 0.989 for LMBV (Fig. S4C).

Our preliminary experiments demonstrated that FV3 could be successfully rescued from naked DNA using LMBV as a helper virus. However, eliminating LMBV after the initial FV3 reconstitution posed a significant challenge. To address this, we developed a quantitative PCR (qPCR) screening system capable of distinguishing between the FV3 and LMBV genomes within the same sample. The qPCR assays were designed to target the major capsid protein (MCP) gene of FV3 (108 bp fragment) and LMBV (62 bp fragment). We confirmed the high specificity of qPCR assays, as they showed no cross-reactivity between FV3 and LMBV (Fig. 3A; Fig. S4A). We further validated the specificity of the assay by testing samples containing FV3 and LMBV DNA, which were combined in reversed serial dilutions. The results demonstrated reliable detection and accurate discrimination between the two viruses (Fig. 3B; Fig. S4B and C).

**Fig 4:**
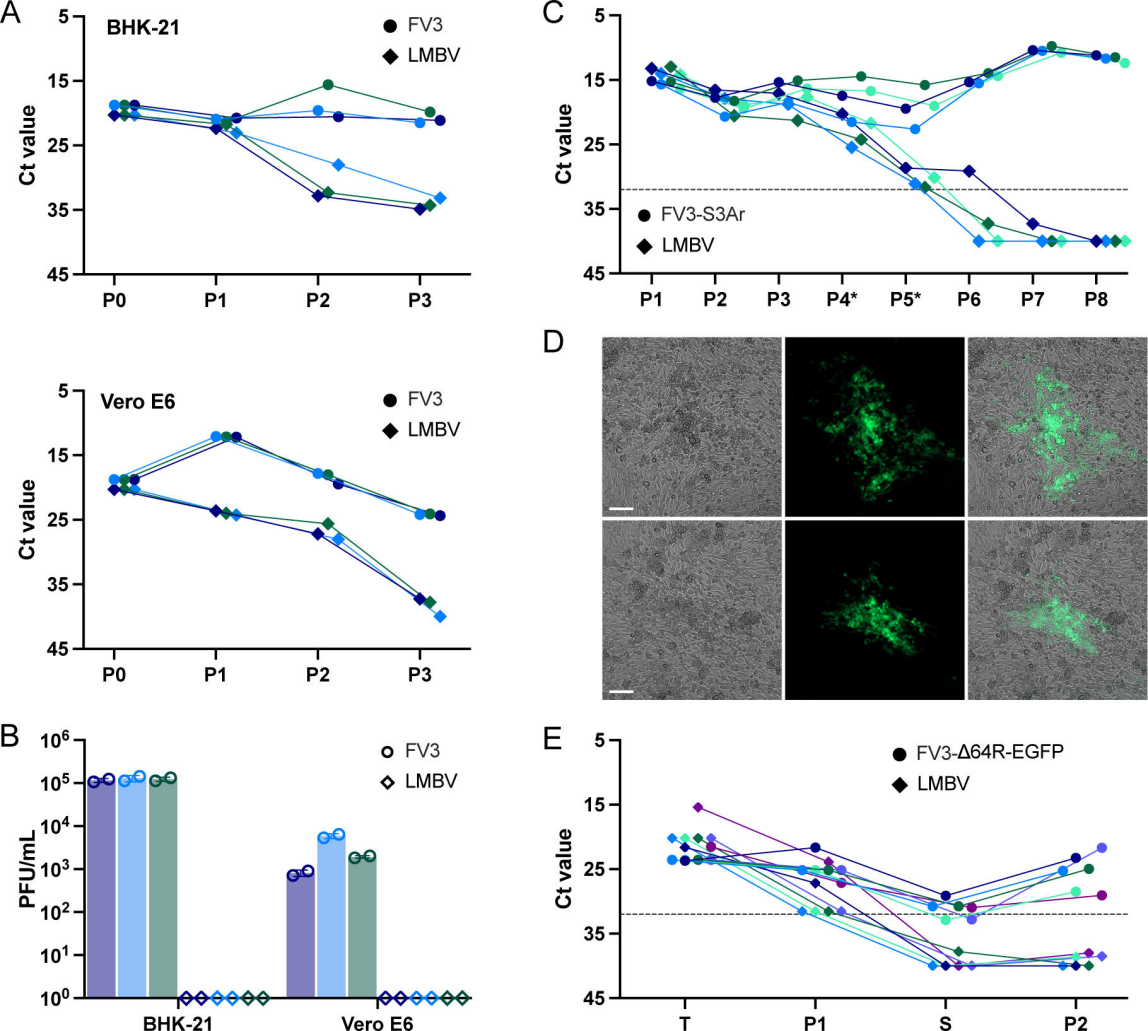
Rescue of FV3-S3Ar and FV3-Δ64R-EGFP using LMBV as a helper virus. (**A**) Replication of FV3 and LMBV on mammalian kidney cell lines BHK-21 and Vero E6. Cells were infected in triplicate at a multiplicity of infection (MOI) of 0.1 with either FV3 or LMBV, and viruses were serially passaged three times to assess their replication potential. Viral genome copy number was determined by qPCR and is shown as mean Ct values from two duplicate measurements. Different colors represent three biological replicates. (**B**) Virus titers from the third virus passage are shown in A. FV3 titers were determined by plaque assay on BHK-21 cells and LMBV titers were determined on BF-2 cells, with duplicate measurements. (**C**) Elimination of LMBV after FV3-S3Ar rescue from bacterial DNA. To remove LMBV, the rescued FV3-S3Ar was initially passaged three times on BHK-21 cells, then twice on Vero E6 cells (labeled as P4* and P5*), and subsequently three times on BHK-21 cells. The graph shows mean Ct values across all passages, determined by duplicate qPCR measurements. Different colors represent four independent biological replicates. The dashed line at Ct 32 indicates the LMBV detection threshold. (**D**) CPE induced by FV3-Δ64R-EGFP in BHK-21 cells 5 days after reconstitution with LMBV helper virus. The left, middle, and right panels show bright-field, fluorescent, and merged images, respectively. Bar, 0.1 mm. (**E**) Elimination of LMBV after FV3-Δ64R-EGFP rescue via cell sorting. FV3-Δ64R-EGFP was rescued in transfected BHK-21 cells using LMBV as a helper virus. After rescue, FV3-Δ64R-EGFP was propagated in uninfected BHK-21 cells. Subsequently, individual cells showing green fluorescence were sorted and seeded onto fresh cells. Finally, FV3-Δ64R-EGFP, originating from sorted cells, was further amplified by an additional passage. The graph shows Ct values for FV3 and LMBV, determined by qPCR at the following stages: T—transfected cells, P1—passage after rescue, S—cells infected with sorted cells, and P2—second passage after sorting. Initially, FV3-Δ64R-EGFP was rescued in three independent events (transfected wells), and the different colors represent six independent FV3-Δ64R-EGFP purifications. The dashed line at Ct 32 indicates the LMBV detection threshold.

Since both FV3 and LMBV can infect a wide range of vertebrate cell lines, we aimed to identify a cell line that supports FV3 while restricting LMBV replication. To achieve this, we infected 10 different cell lines (BHK-21, MRC-5, RK-13, Vero E6, CCO, MDCK, T7, CEC, ICR-2A, and A6) with either FV3 or LMBV and transferred 5% of the cell culture medium to the next passage every 2 days. We assessed the presence of each virus over the course of passaging by qPCR (Fig. S5A). From the tested cell lines, BHK-21 cells emerged as the best-suited for the elimination of LMBV, as they efficiently supported FV3 propagation (46) while restricting LMBV replication (Fig. 4A). Vero E6 cells also showed reduced LMBV replication, though FV3 propagation was less efficient compared to BHK-21 cells.

When we used BHK-21 cells for LMBV-aided FV3 rescue, we observed that LMBV was not completely eliminated from the rescued FV3 (clone S3A) over the course of passaging on the BHK-21 cells (Fig. S5B). We speculate that active FV3 replication might help maintain a low level of LMBV replication in BHK-21 cells. To overcome this issue, we added an intermediate step involving passage of the rescued FV3 on Vero E6 cells. We selected Vero E6 cells based on our initial screening (Fig. S5A) and subsequent experiments, which demonstrated that while these cells strongly restrict LMBV replication, they also moderately restrict FV3 replication (Fig. 4A). While FV3 replicates rapidly in many different cell lines, it takes 4 to 5 days for FV3-induced plaques to appear on Vero E6 cells (Fig. 2B and C). By incorporating this passage on Vero E6 cells, we successfully eliminated LMBV, which we confirmed by titration of the final passage on BF-2 cells (Fig. 4B).

Our final, optimized procedure to recover FV3 (FV3-S3Ar) from bacterial DNA using LMBV as a helper virus involved several steps. First, we infected BHK-21 cells with LMBV for 2 h and then transfected them with purified FV3 DNA. To increase the titer of the rescued virus, we collected the cell culture medium from the transfected cells 2 days after transfection and used it to infect fresh BHK-21 cells. We repeated this passage on BHK-21 cells two more times, with each infection lasting 2 days. After this initial amplification of the virus, we eliminated LMBV by two consecutive passages on Vero E6 cells, with each infection also lasting 2 days. We opted for two passages on Vero E6 cells because previous attempts with a single passage on Vero E6 cells did not fully eliminate the presence of LMBV (Fig. S5C). Following passaging on Vero E6 cells, we performed three additional virus passages on BHK-21 cells and confirmed complete eradication of LMBV replication by qPCR (Fig. 4C*;* Fig. S5D).

In addition, we performed NGS on virus harvested from the last three virus passages on BHK-21 cells (after the passage on Vero E6 cells) and detected no apparent signs of recombination between FV3 and LMBV. Instead, sequencing confirmed the expected presence of an intact FV3 genome. Overall, the rescue of FV3 from purified bacterial DNA with a heterologous fish helper virus provides an elegant and efficient way to recover FV3. In our hands, this procedure also resulted in a more homogeneous plaque phenotype compared to attempts using UV-irradiated virus and circumvented any necessity for plaque purification. In addition, using the least similar ranavirus as a helper virus decreases the risk of undesirable recombination events between the rescued FV3 and the helper virus.

To simplify and accelerate the elimination of LMBV after rescue, we explored cell sorting as an alternative method to purify FV3. Since this approach typically requires the purified virus to express a fluorescent protein, we tested it using FV3-Δ64R-EGFP. We rescued FV3-Δ64R-EGFP from bacterial DNA using LMBV as a helper virus in three independent experiments (Fig. 4D). The rescued virus was then passaged on fresh BHK-21 cells, and after 5 days, these cells were subjected to cell sorting. During sorting, single green fluorescent cells were transferred to wells of a 48-well plate containing BHK-21 cells. After 2 days, approximately 10% of the wells exhibited green fluorescence. The rescued FV3-Δ64R-EGFP was then passaged once more on BHK-21 cells and tested for the presence of LMBV by qPCR (Fig. 4E). None of the tested samples contained LMBV, confirming that cell sorting is an efficient method for eliminating LBMV helper virus and purifying fluorescently labeled FV3.

### Characterization of the rescued virus

To evaluate the growth properties of BAC-YAC-derived FV3, we assessed the multi-step growth kinetics of three viruses that were independently rescued from the S3Ar clone using the UV-irradiated FV3-ΔORF64R-EGFP as a helper virus (Fig. 1D). Alongside, we evaluated the multi-step growth kinetics of four independently rescued viruses from the S3Ar clone using the LMBV as a helper virus (Fig. 1E). Growth kinetics experiments were performed on BHK-21 cells at multiplicity of infection (MOI) of 0.005, and the growth of the S3Ar viruses was compared to that of the parental FV3 isolate (FV3-FUB, Fig. 1D and E). No significant differences were detected in viral replication kinetics between the parental FV3 and S3Ar-derived viruses, regardless of whether they were rescued using the UV-irradiated FV3 or LMBV helper virus. However, virus rescues with LMBV exhibited slightly higher titers compared to those rescued with the UV-irradiated virus.

To assess the genetic stability of BAC-YAC-derived FV3-S3A and FV3-ΔORF64R-EGFP viruses, we passaged the rescued viruses 10 times serially on BHK-21 cells. For each passage, 5% of the cell culture media from the existing passage was used as inoculum for the next passage. The passaging was done every 2 days in triplicate. Sequencing of virus genomes from passage ten did not detect any sequence changes in any of the passaged viruses, suggesting high genetic stability of both the rescued WT-like and recombinant EGFP viruses. In accordance with these results, the FV3-ΔORF64R-EGFP maintained strong EGFP expression throughout the passaging experiment.

## DISCUSSION

To our knowledge, this study is the first to develop a reverse genetics system for a key member of the class *Megaviricetes*. Harnessing the power of TAR cloning in yeast, we generated a recombinant BAC-YAC clone containing the entire copy of the FV3 genome. The virus rescued from this system exhibited a cell culture phenotype that was indistinguishable from the parental FV3 isolate. Reverse genetics systems are invaluable tools in molecular virology, as they allow genetic manipulation of viral genomes in bacteria, eliminating the need for tedious and time-consuming mutagenesis procedures based on recombination in eukaryotic cells (47). To demonstrate this important improvement, we utilized our synthetic infectious clone to generate a reporter virus. Specifically, we replaced ORF64R, encoding a known virulence factor, with EGFP, similarly as described in a previous study (20). Our bacterial FV3 clone, combined with the two-step Red recombination-based mutagenesis procedure, known as “*en passant”* mutagenesis, enables the generation of FV3 mutants within less than 2 weeks (22, 28). Moreover, the recombinant EGFP virus could be repeatedly reconstituted in cell culture, but it also showed impeccable genetic stability when passaged in permissive host cells. This reverse genetics system represents a pivotal advance in iridovirus research, as it streamlines the manipulation of viral genomes with remarkable efficiency and speed.

The inability of iridoviruses to form replicating progeny following the transfection of purified DNA in cell lines permissive for viral replication presents a considerable barrier to the practical application of reverse genetics systems. Although the use of irradiated homologous helper virus for rescuing replicating virus from DNA has long been described, we have confirmed that irradiated helper virus must retain some replicative capacity (39) and warrants further research. This could be problematic both in terms of potential contamination of stocks and recombination between the helper and the rescued viruses (48, 49). Therefore, we set out to find an alternative approach to FV3 rescue from DNA using LMBV as a heterologous helper virus. LMBV is genetically distinct from FV3, as a nucleotide sequence alignment using discontiguous megaBLAST (50) revealed that FV3 (GenBank: NC_005946.1) and LMBV genomes (GenBank: MW630113.1) only aligned about 37% of the genomes, with a nucleotide identity of approximately 72% within these regions. These genetic differences lower the risk of unwanted recombination between the two genomes during the rescue process. Yet, the LMBV protein(s) that are presumed essential for FV3 rescue appear to exhibit sufficient similarity to FV3 proteins to aid the process. Thus, the use of LMBV as a helper virus provides a convenient and highly reproducible procedure for FV3 rescue.

The use of reverse genetics systems for easy and efficient virus mutagenesis holds great promise for research to better understand many yet enigmatic aspects of iridovirus biology. However, beyond this, FV3 with its ability to infect a wide range of poikilotherm hosts (4) and to efficiently replicate in cell lines derived from mammals, amphibians, and fish has considerable potential for vaccine development. Our system allows the incorporation of any desired gene of interest into the FV3 genome, as well as the deletion or replacement of specific virulence factors. Employing such an approach enables the rational design of modified live vaccines, a strategy previously applied in other virus families, such as herpesviruses or poxviruses (51–53). This research may be particularly important considering the ongoing sixth mass extinction (15, 54, 55), with amphibians suffering dramatic losses associated with infectious diseases (56–59). The development of effective vaccines against iridoviruses and other amphibian pathogens could play a crucial role in conservation efforts, potentially mitigating the impact of these pathogens on vulnerable populations.

## MATERIALS AND METHODS

### Cells and viruses

Baby hamster kidney cells (BHK-21, ATCC_CCL-10), primary chicken embryo cells (CEC), Madin-Darby canine kidney cells (MDCK, ATCC_CCL-34), rabbit kidney epithelial cells (RK13; ATCC_CCL-37), and African green monkey kidney (Vero E6; ATCC_CRL-1586) cells were grown in Dulbecco’s modified Eagle’s medium (DMEM; PAN-Biotech) supplemented with 5% fetal bovine serum (FBS; PAN-Biotech), 100 units/mL of penicillin and 100 µg/mL of streptomycin (1% P/S, Thermo Fisher Scientific). Medical Research Council Cell Strain-5 (MRC-5; ATCC_CCL-171) cells and T7 cells (60) were grown in DMEM supplemented with 20% FBS and 1% P/S. BHK-21, MDCK, RK13, Vero E6, MRC-5, and T7 cells were incubated at 37°C with 5% CO_2_. Fish cell lines, including BF-2 cells (ATCC_CCL-91), derived from the bluegill (*Lepomis macrochirus*) and CCO cells, derived from brown bullhead (*Ameiurus nebulosus*) were cultured in D1152 high glucose medium (Sigma-Aldrich; pH 7.4) supplemented with 10% Heat Inactivated FBS (Gibco) and 1% P/S at 23°C without CO_2_. The amphibian cell line A6 (ATCC_CCL-102), derived from African clawed frog (Xenopus laevis), was cultured in IMDM medium (PAN-Biotech) diluted with 30% sterile distilled water, supplemented with 10% FBS and 1% P/S. Amphibian haploid cell line ICR-2A (ATCC_CCL-145), derived from northern leopard frogs (*Lithobates pipiens*), were cultured in Ham’s F12 medium (Sigma-Aldrich) diluted with 40% sterile distilled water, supplemented with 10% FBS, 1% P/S, 1.76 g/L sodium bicarbonate, and 10 µg/mL voriconazole. Both amphibian cell lines were cultured at 25°C in a 5% CO_2_ incubator.

FV3, kindly provided by Jacques Robert, was propagated in confluent monolayers of BHK-21 cells incubated at 30°C during infection (61). To produce FV3 stocks, BHK-21 cells were infected at an MOI of 0.03, and cell culture medium was harvested 5 days after infection, when complete cell lysis was observed. The type strain of LMBV (40), generously provided by Larry Hanson, was propagated on confluent monolayers of BF-2 cells and incubated at 23°C for 3 to 4 days until most cells were lysed. To prepare virus stocks, FV3- or LMBV-infected cells along with infectious medium were frozen at −80°C and subsequently thawed at room temperature. Cellular debris was pelleted by centrifugation at 1,800 × *g* for 15 min at 4°C (46), and the clarified supernatant was collected and stored at −80°C. Virus titers were determined by plaque assays.

### Testing FV3 and LMBV replication in different cell lines

To assess the replication capacity of FV3 and LMBV in different cell lines, BHK-21, MRC-5, RK-13, Vero E6, CCO, MDCK, T7, CEC, ICR-2A, and A6 cells were grown in T-25 flasks with 5 mL of medium. Cells were infected at an MOI of 0.1 with either FV3 or LMBV. The cell culture medium was replaced after 24 h. After 2 days of incubation, the virus was passaged by transferring 5% of the infectious medium (250 µL) to fresh cells. The presence of viral genome copies was monitored throughout the passages using qPCR. The number of infectious virus particles in passage was determined by plaque assay on permissive cells (BHK-21 cells for FV3 and BF-2 cells for LMBV). Since BHK-21 and Vero E6 cells were identified as the most optimal for removing LMBV (supporting FV3 replication while limiting or excluding LMBV replication), we repeated the passaging experiment with FV3 and LMBV on these cells a total of three times.

### Plaque assay

Virus titration for FV3 and LMBV was performed as described (46), using 10-fold serial dilutions in DMEM supplemented with 5% FBS. Serial virus dilutions were added in duplicates to 90% confluent BHK-21 cells (for FV3) or BF-2 cells (for LMBV), grown in 6-well plates. The plates were incubated at room temperature for 1 h with constant gentle rocking to ensure even distribution of the virus. After the incubation, the inoculum was removed, and cells were overlaid with 3 mL of 1.5% methylcellulose in DMEM with 5% FBS. The plates were then incubated for 7 days at 30°C for FV3 and at 30°C for LMBV. To visualize plaques, the cells were fixed with 4% formaldehyde in phosphate-buffered saline (PBS) and stained with 0.75% crystal violet aqueous solution. Images of the plaques were captured at 25× magnification using an inverted microscope Zeiss Axiovert S100. Plaque areas were measured using ImageJ (62), from which, assuming that ideal plaques would have a circular shape, plaque diameters were calculated and are presented in arbitrary units (a.u.).

### FV3 fragment preparation, TAR cloning, and clone screening

FV3 DNA was isolated from infected BHK-21 cells using the sarcosine lysis buffer method (63). To enable the genome assembly, we designed primers to amplify the entire FV3 genome (105,903 bp) as 14 overlapping fragments, each approximately 8 kb in size (Fig. S1). The overlapping regions between adjacent fragments were designed to be approximately 100 to 300 bp long (Fig. S1). Additionally, the first and the last FV3 fragments had an overlap for the BAC-YAC vector. The BAC-YAC vector harbors essential components for replication in both bacterial and yeast systems, including the origin of replication oriS for propagation in *E. coli*, and autonomously replicating sequence-like sequences and a centromere, required for replication in *S. cerevisiae* (64, 65). To minimize the introduction of unwanted mutations into the cloned FV3 genome, fragments were amplified using a high-fidelity PCRBIO VeriFi Polymerase (PCR Biosystems), following the conditions described in Table S3. The resulting PCR products were purified from agarose gels using the Monarch PCR & DNA Cleanup Kit (NEB). TAR cloning was performed exactly as described previously (27). DNA was isolated from several yeast clones using the zymolyase lysis method (27) and electroporated into *E. coli* (E. cloni 10G BAC-Optimized Electrocompetent cells; LGC Biosearch Technology). Next, DNA was isolated from several bacterial clones and screened by RFLP analysis using *Hind*III*, Apa*LI, and *Sal*I restriction enzymes to identify correctly assembled constructs.

### Optimization of UV-irradiation conditions

UV irradiation was performed using a 30-Watt germicidal UV lamp (TUV 30W/G30 T8, Philips) on 5,000,000–7,000,000 PFU of FV3 suspended in 1 mL DMEM in a 60 mm² tissue culture dish. To determine the optimal irradiation conditions, we tested various exposure times (3, 9, 12, 15, 20, and 30 min) and distances between the UV lamp and the cell culture dish containing the virus (10, 20, 30, 40, 50, 60, and 70 cm). We observed that when the virus was irradiated at distances shorter than 70 cm, it was over-irradiated and could not be used as a helper virus. BHK-21 cells grown to 90% confluency in 6-well plates that were exposed to 100 µL of the UV-irradiated virus died within 24 h, regardless of the exposure time. Therefore, we standardized the distance at 70 cm. Further testing revealed that UV irradiation at 70 cm for 3, 9, 12, or 15 min successfully enabled FV3 rescue from DNA, while the virus exposed to UV for more than 15 min failed to recover FV3. Thus, a 15 min exposure time at a distance of 70 cm was selected as the optimal irradiation condition for rescuing FV3 using UV-irradiated helper virus.

### Rescue of FV3 from BAC-YAC DNA with UV-irradiated helper virus

For the rescue of FV3 from BAC-YAC constructs, we used either UV-irradiated FV3-FUB or UV-irradiated FV3-ΔORF64R-EGFP helper virus. BHK-21 cells were grown to 90% confluency in 6-well plates. The growth medium was aspirated, and the cells were incubated with UV-irradiated helper virus at an MOI of 1 (extrapolated from batch measurements before UV-irradiation) in 500 µL medium for 2 h at 30°C with 5% CO_2_. After incubation, the helper virus was removed, and cells were overlayed with 2 mL of DMEM containing 5% FBS. Next, FV3 BAC-YAC DNA was transfected using Lipofectamine 3000 (Thermo Fisher Scientific), following the manufacturer’s instructions. Importantly, we observed that FV3 irradiated under the optimal conditions retained a reduced replication capacity. The UV-irradiated virus still caused CPE on BHK-21 cells, albeit much later than the WT virus. CPE was first observed approximately 2 weeks post-infection, in contrast to the more rapid onset seen with WT FV3.

### Rescue of FV3 BAC-YAC DNA with LMBV

The rescue of FV3 BAC-YAC clones with LMBV as a heterologous fish helper virus was identical to the rescue procedure with the UV-irradiated virus described above, with the exception that live LMBV was used at an MOI of 0.5. After successful rescue, FV3-S3A or FV3-S3Ar were passaged three times on BHK-21 cells, followed by two passages on Vero E6 cells and an additional three passages on BHK-21 cells.

### Cell sorting

FV3-Δ64R-EGFP was independently rescued three times using LMBV helper virus. Two days after the transfection, the rescued virus was passaged onto BHK-21 cells grown in 75 cm² tissue culture flasks. The cells were incubated at 30°C with 5% CO₂ for 5 days, by which time a significant number of cells exhibited strong green fluorescence, indicating FV3-Δ64R-EGFP infection. The cells were then trypsinized, collected by centrifugation (250 × *g*, 5 min, RT), and washed twice with PBS. The pelleted cells were resuspended in 2 mL of FACS buffer (PBS supplemented with 0.2% bovine serum albumin [BSA] and 2 mM EDTA) at a final concentration of approximately 10 million cells per mL. Subsequently, fluorescent cells were sorted using a FACSAria II sorter (BD Biosciences). Individual fluorescent cells from each independent virus rescue were transferred into 48-well plates containing BHK-21 cells. The plates were then incubated at 30°C with 5% CO_2_ for 3 days. The presence of green fluorescence was observed under a fluorescent microscope, and the number of wells showing a strong fluorescent signal was recorded. FV3-Δ64R-EGFP from six selected wells, two from each rescue, was passaged 3 days after sorting by transferring 200 µL of the cell culture medium onto fresh BHK-21 cells. The cells were then incubated for 3 more days, and the presence of FV3 and LMBV was assessed by qPCR.

### Generation of EGFP reporter virus

To generate a reporter virus from the obtained FV3 BAC-YAC, we replaced the virulence gene Δ64R (nucleotide position 75.818–76.105 in the parental sequence), which encodes a putative interleukin-1 beta convertase precursor, with the coding sequence of EGFP. We chose this gene as previous studies have already shown that its deletion does not affect the FV3 virus growth on a mammalian cell line (20). The replacement was performed by *en passant* mutagenesis (38). The accuracy of the replacement Δ64R gene with EGFP was confirmed by sequencing the mutated region, as well as through whole genome sequencing of the FV3-Δ64R-EGFP viral mutant.

### Multistep-growth curve analysis

BHK-21 cells were grown to 80% confluency in 6-well plates (Sarstedt) and infected in duplicate with three independent rescues of S3Ar using UV-irradiated virus and four independent rescues of S3Ar using LMBV at MOI of 0.005. The plates were incubated at 30°C with 5% CO_2_. Two wells infected with each virus were immediately harvested at 0 h time points and frozen at –80°C, with subsequent time points collected in duplicate at 12, 24, 48, 72, and 96 h after infection. Virus titers for each time point were determined by plaque assay. Growth kinetics were performed with passage 2 virus resulting from rescue with UV-irradiated virus and passage 6 after rescue with LMBV. Graphs and statistical analysis were performed using GraphPad Prism V10.4.1. Standard one-way analysis of variance tests were used to assess statistical differences.

### Immunofluorescence staining

For the immunofluorescence assay, confluent BHK-21 cells grown in 6-well plates were infected with the rescued clone S3Ar for 5 days. Cells were fixed with 4% paraformaldehyde, permeabilized with 0.1% Triton X-100 in PBS for 10 min, and then blocked with 3% bovine serum albumin (BSA; Carl Roth) in PBS for 1 h. The blocking solution was removed, and 500 µL murine mAb anti-EHNV 7N9A (kindly provided by Sven Reiche, Friedrich-Loeffler-Institut, Insel Riems) in 3% BSA/PBS was added to each well. The plates were incubated with the primary antibody for 1 h then washed three times with PBS. Subsequently, 500 µL of secondary antibody (goat anti-mouse IgG Alexa Fluor 647; Thermo Fisher Scientific) conjugated with a far-red fluorescent dye and diluted 1:3,000 in 3% BSA/PBS was added to each well. The plates were incubated for 1 h and then washed three times with PBS. Stained cells were observed using a fluorescence microscope (Zeiss).

### DNA extraction and qPCR

Viral FV3 BAC-YAC DNA was extracted from 200 µL of infected cell culture supernatant using the innuPREP Virus DNA/RNA Kit (IST Innuscreen). Briefly, 200 µL of infected supernatant was mixed with lysis buffer (including Carrier Mix) and digested using Proteinase K. DNA was purified using a spin column procedure and eluted in 60 µL of nuclease-free water. Fluorometric (Qubit, Invitrogen) DNA concentration for eluates typically ranged between 10 and 20 ng/µL. Extracted DNA was stored at −20°C until further analysis. For the qPCR, we used conditions that were previously described (66): 2 min at 50°C, 10 min at 95°C for activation of the enzyme, and 40 cycles of 15 s at 95°C and 60  s at 60°C on a qTower G3 cycler (Analytic Jena) in sealed qPCR 96-well plates. qPCR was performed using the SensiFAST Probe Lo-ROX (BioCat) reagent. For one qPCR reaction, 10 µL of SensiFAST MasterMix (2×), 0.9 µL (400 nM) of forward primer, 0.9 µL (400 nM) of reverse primer, 0.2 µL (250 nM) of a probe targeting the MCP portion of FV3 or LMBV, 3 µL of DEPC water (Carl Roth), and 5 µL of DNA template were used. The primer and probe sequences for the amplification of LMBV (67) and FV3 are provided in Table S4. Simultaneous qPCR for FV3 and LMBV was specific, and the virus detection lacked cross-reactivity with each other. The detection threshold for qPCR was set at 32 Ct after experimental determination: DNA was isolated from uninfected BHK-21 cells and checked for a positive qPCR signal. Linear regression analysis for qPCR data of FV3 and LMBV was conducted using R (45).

### Sequencing

Sequencing of FV3 BAC-YAC clones and of the isolate was performed using next-generation Illumina sequencing. Sequencing libraries were prepared using the NEBNext Ultra II DNA Library Prep Kit (NEB). DNA fragmentation was done using the Covaris M220 focused-ultrasonicator on 1–5 μg of extracted diluted up to 130 µL 0.1× TE Buffer. The size selection step of the NEBNext Ultra II DNA Library Prep protocol was included for the selection of fragments between 500 and 700 bp. All other steps were performed as per the manufacturer’s instructions. The final enriched libraries were pooled and loaded into the MiSeq instrument as per Illumina’s instructions. The resulting data were processed with Trimmomatic v.0.39 (68) and mapped against a reference FV3 genome (GenBank accession number: NC_005946.1) using the Burrows-Wheeler aligner v.0.7.17 (69). Mapping statistics were generated using Samtools v1.10 (70), and the resulting alignment was visualized and inspected using IGV v2.9.4 for Linux (71). For the detection of SNPs and other variants, a Bayesian genetic variant detector (Freebayes) (72) was used with the following parameters: only SNPs with a minimum mapping quality of 5, minimum count of 3, and minimum fraction of 0.01 were considered. Consensus sequences were then obtained using BCFtools (70). Raw sequencing data is available at the Sequencing Read Archive (SRA) under BioProject number PRJNA1156356. The pre-processing, mapping, and variant-calling steps are wrapped in a custom script (available at https://github.com/mmnascimento/FV3). The obtained consensus sequence is available in the aforementioned GitHub repository and at DNA Data Bank of Japan (DDBJ) under accession number LC830689.

In addition to this, we sequenced bacterial clones FV3-S3A and FV3-ΔORF64R-EGFP using Oxford Nanopore sequencing technology (Plasmidsaurus).

## Data Availability

The FV3 BAC-YAC genome, clone S3A, and sequence data reported in this article have been deposited in NCBI/ENA/DDBJ (LC830690). Raw sequencing data are available at the Sequencing Read Archive (SRA) under BioProject number PRJNA1156356. Scripts used for data analysis, as well as the obtained consensus sequences, are available at https://github.com/mmnascimento/FV3.

## SUPPLEMENTAL MATERIAL

Supplemental material jvi.01852-24-s0001.pdf Figures S1 to S5 and Tables S1 to S4.

ASM does not own the copyrights to Supplemental Material that may be linked to, or accessed through, an article. The authors have granted ASM a non-exclusive, world-wide license to publish the Supplemental Material files. Please contact the corresponding author directly for reuse.
