# Supplementary material for: Engineering iridoviruses: development of reverse genetics and virus rescue systems
Source: J Virol. 2025 Apr 17;99(5):e01852-24. doi: 10.1128/jvi.01852-24 (PMC12090718; doi:10.1128/jvi.01852-24)
Supplement: Supplemental material — Figures S1 to S5 and Tables S1 to S4. [file jvi.01852-24-s0001.pdf]

Supporting Information for

Engineering iridoviruses: Development of reverse genetics and virus rescue systems

Daria Vladimirova, Daniela Kunecova, Mariana Nascimento, Ji Yoon Kim, Dusan Kunec and Jakob Trimpert

Correspondence to: Jakob Trimpert
Email: **jtrimpert@vet.k-state.edu**.

This PDF file includes:

Figures S1 to S5
Tables S1 to S4

Figure S1.

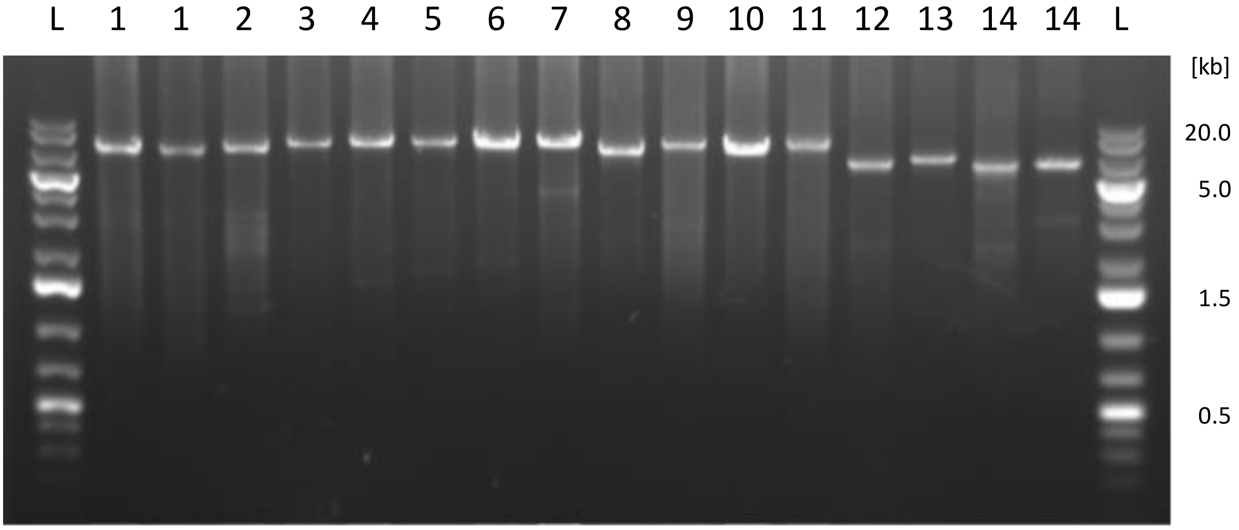

Fig. S1. Overlapping FV3 fragments 1 to 14 used for the assembly of BAC-YAC clone of FV3 by TAR cloning. The amplified fragments were purified from the gel and used for TAR cloning. Fragment 1 and fragment 14 were run in two lanes. L,1 Kb Plus DNA ladder (Thermo Fisher Scientific).

Figure S2.

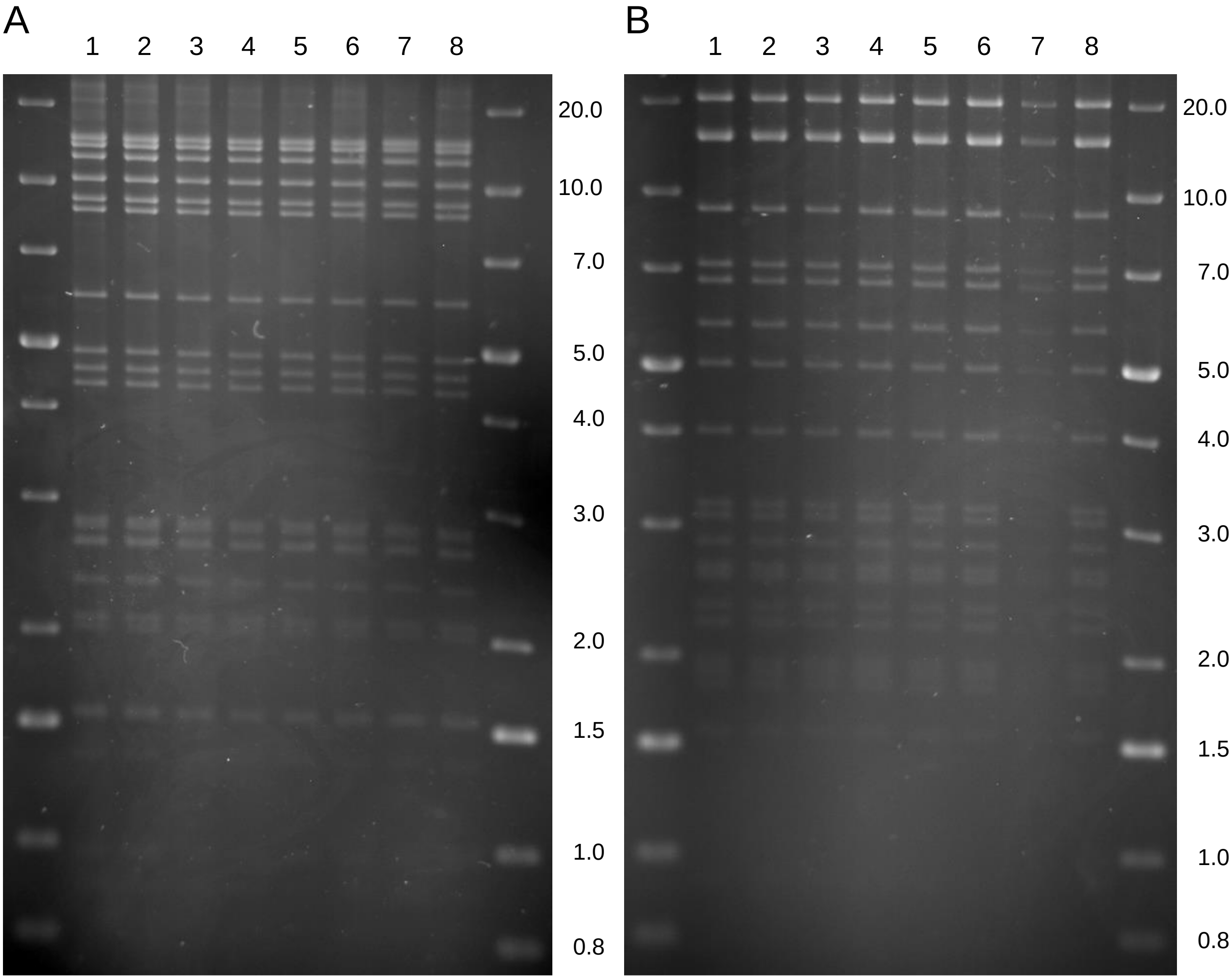

Fig. S2. RFLP analysis of FV3 BAC-YAC clones. (**A**) RFLP analysis with ApaLI. Restriction fragments (bp): 13,645, 12,959, 11,359, 10,107, 8,978, 8,506, 5,813, 4,765, 4,479, 4,268, 2,785, 2,722, 2,626, 2,606, 2,320, 2,070, 1,992, 1,550, 1,541, 1,527, 1,333, 971, 959, 852, 738, 607, 580, 381, 313, 294, 128, 93, 26 (**B**) RFLP analysis with SalI. Restriction fragments (bp): 20,279, 14,117, 13,817, 8,920, 6,972, 6,573, 5,593, 4,893, 3,954, 3,139, 3,044, 2,812, 2,605, 2,529, 2,306, 2,189, 1,936, 1,881, 1,795, 1,539, 884, 657, 563, 521, 375. Lanes 1 to 8: BAC-YAC clones R1A, R1B, R2A, R2B, S1A, S2A, S3A, and S4A. DNA marker: 1 Kb Plus DNA ladder (Thermo Fisher Scientific).

Figure S3.

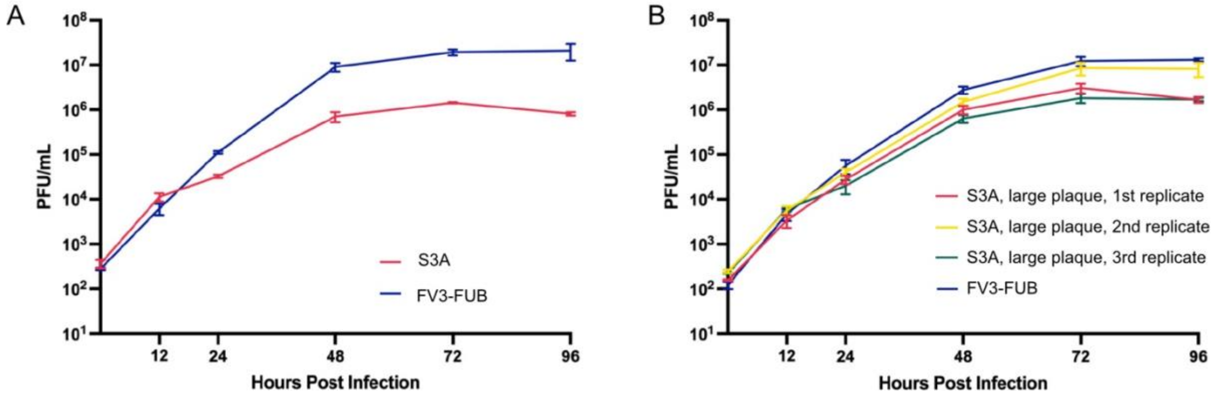

Fig. S3. FV3 rescue from the BAC-YAC clone S3A using LMBV as a helper virus. (***A, B***) The replication of the S3A virus, which carries the A72T mutation in ORF85R, and the parental isolate FV3-FUB was assessed by growth kinetics on BHK-21 cells in two biological replicates. Virus titers were determined by plaque assay in two technical replicates. The mean virus titers are shown as PFU/mL, and error bars represent the standard deviation (SD). The S3A virus exhibited significantly reduced replication compared to the parental FV3-FUB, with titers nearly 100-fold lower. However, the repaired S3Ar virus exhibited growth properties similar to the parental virus (Fig. 1D and E). (***B***) Growth properties of three S3A virus isolates (ORF85R, A72T) after purification of viruses from larger plaques.

Figure S4.

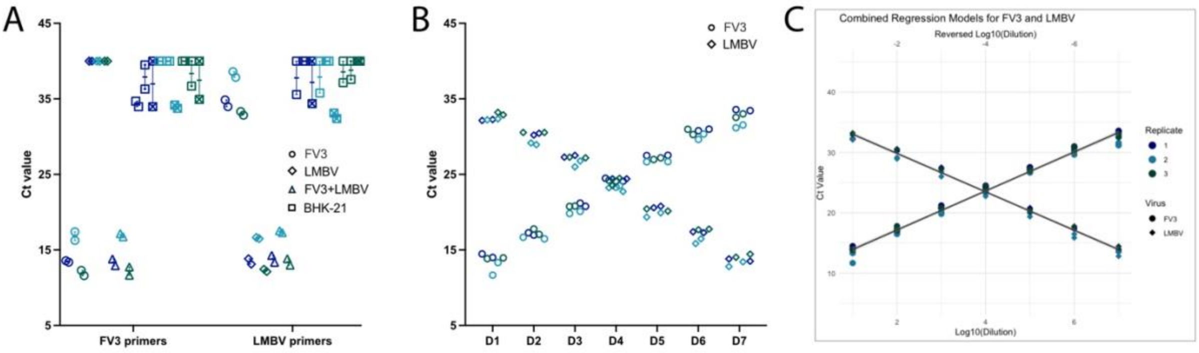

Fig. S4. Validation of qPCR assays for detection of FV3 and LMBV sequences (expanded from Fig. 3). (***A***-***B***) Ct value measurements were performed in biological triplicates and technical duplicates. Replicate one is shown in blue, replicate two in light blue, and replicate three in green. (***A***) To evaluate the specificity of qPCR assays for discriminating FV3 (circles) and LMBV (diamonds), assays were performed on samples containing only FV3 or LMBV DNA, as well as on mixed samples containing DNA from both viruses (triangles). Negative controls containing DNA from uninfected BHK-21 cells were run in 9 replicates (square, square with a dot, crossed square). Both qPCR assays demonstrated high target specificity, with FV3 detection showing slightly higher specificity. Since, in one sample, DNA extracted from uninfected BHK-21 cells yielded Ct values of 32.4 and 33.1 with the LMBV primer set, a Ct value of 32 was set as the detection threshold. The data are plotted as individual values, with the mean and the range indicated. (***B***) The cross-reactivity of the qPCR assays for FV3 (circles) and LMBV (diamonds) was assessed using samples containing varying ratios of both viral genomes (D1: FV3:LMBV as 1000 ng: 0.001 ng, D2: 100 ng: 0.01 ng, D3: 10 ng: 0.1 ng, D4: 1 ng: 1 ng, D5: 0.1 ng: 10 ng, D6: 0.01 ng: 100 ng, D7: 0.001 ng: 1000 ng). The data are plotted as individual values. (***C***) The linearity (R^2) of the pooled biological replicates in technical duplicates, calculated using R [66], reached 0.987 for FV3 and 0.989 for LMBV.

Figure S5.

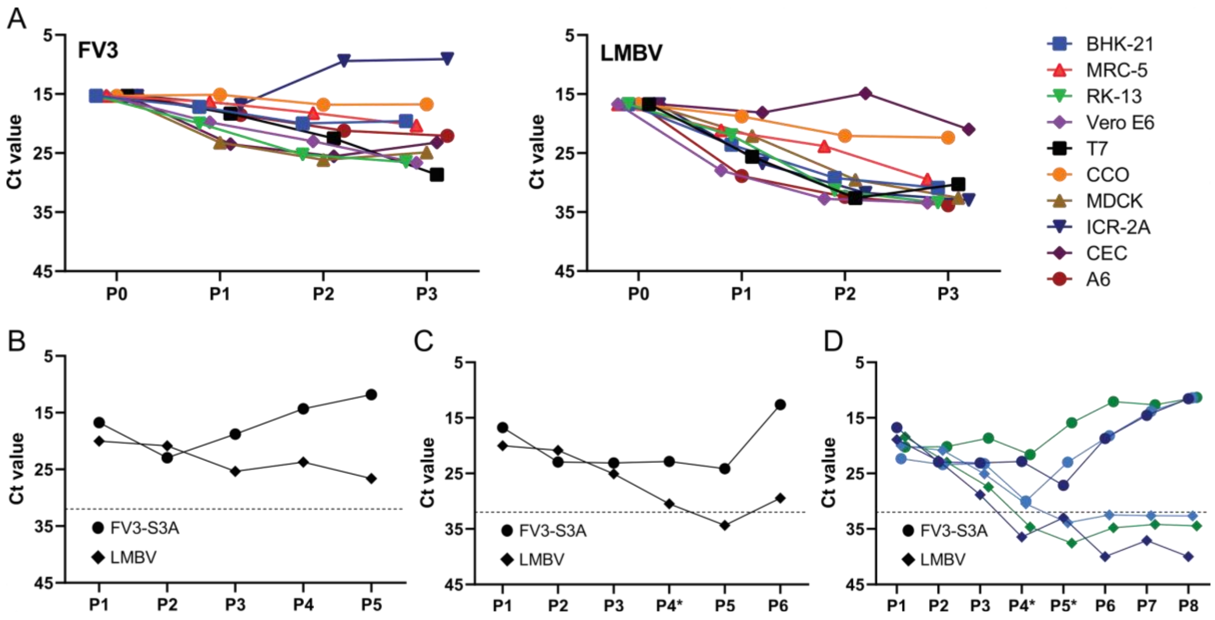

Fig. S5. Evaluation of FV3 and LMBV propagation in different cells lines and elimination of LMBV after FV3-S3A rescue. (***A***) Assessment of the replication capacity of FV3 and LMBV across various cell lines. BHK-21, MRC-5, RK-13, Vero E6, CCO, MDCK, T7, CEC, ICR-2A, and A6 cells were infected with either FV3 or LMBV at an MOI of 0.1. The graphs show the average Ct values for each passage, measured in duplicate. (***B–D***) Quantification of FV3-S3A and LMBV replication after FV3-S3A rescue using LMBV as a helper virus. The quantities of rescued FV3-S3A (circles) and LMBV (diamonds) were determined by qPCR. The graphs show the mean Ct values for each passage, determined in technical duplicates. The dotted line at Ct 32 indicates the detection threshold. (*B*) Although BHK-21 cells were found to be non-permissive for LMBV replication, LMBV DNA remained detectable in the cell culture medium after five passages. (*C*) A single passage on Vero E6 cells (labelled P4*) significantly reduced LMBV genome copy numbers, but LMBV was not completely eliminated. (*D*) Two consecutive passages on Vero E6 cells (P4* and P5*) successfully eliminated LMBV, as no LMBV DNA was detected in subsequent passages (P6–P8). The graph shows results from three independent biological replicates. The first replicate is shown in dark blue, the second in light blue, and the third in green.

Table S1. Sequence variations between the FV3 reference genome and the FV3 FUB isolate.

No.	Position (NC_005946.1)	Reference (NC_005946.1)	FV3 FUB (LC830689.1)	Mutation	Location	Effect
1	13,742	A	C	A→C	ORF9L	Silent
2	15,187-15,188	GA	-	2-bp deletion	Intergenic	Non-coding region
3	33,571	G	C	G→C	Intergenic	Non-coding region
4	39,144/39,145	-	279-bp sequence	279-bp insertion	ORF32R	ORF extension
5	51,070	G	-	1-bp deletion	ORF43R	ORF extension
6	51,741	A	-	1-bp deletion	Intergenic	Non-coding SNP
7	51,889	G	C	G→C	Intergenic	Non-coding SNP
8	52,455	C	-	1-bp deletion	Intergenic	Non-coding SNP
9	52,611/52,612	-	17-bp sequence	17-bp insertion	Intergenic	Non-coding region
10	52,738	T	-	1-bp deletion	ORF46L	ORF extension
11	54,775	C	-	1-bp deletion	ORF50L	ORF49/50L fusion
12	75,982	A	C	A→C	Intergenic	Non-coding SNP
13	80,902-80,905	CACA	-	4-bp deletion	Intergenic	Non-coding region
14	97,018-97,059	42-bp sequence	-	42-bp deletion	ORF89R	In-frame deletion
15	97,992	C	G	C→G	ORF90R	H216Q
16	102,223	G	C	G→C	ORF95R	V190L
17	104,642	C	G	C→G	ORF97R	L114V

Table S2. Primers to generate FV3 fragments for TAR cloning.

Primers for amplification of overlapping FV3 fragments					
**Fragment**	**Primer**	**Sequence (5'-3')**	**Fragment position (NC_005946.1)**	**Product size (bp)**	**Overlap with next fragment (bp)**
1	DV01	AAG-CTT-TAA-CAG-ATT-CAT-GAA-ATT-GT	1-7,611	7,610	227
	DV02	CGT-CAA-AGA-ACT-TTG-ACA-GC			
2	DV04	CAA-ACA-GGA-GTC-CTA-CTG-C	7,384-15,396	8,012	126
	DV05	AGT-CTT-GAC-ACT-TGT-CAT-GG			
3	DV06	GAG-AGA-GCA-TAT-CCT-GAG-AG	15,270-23,482	8,212	124
	DV07	CAC-CAT-GTT-GTT-TAC-GAC-C			
4	DV08	AGT-TTG-TCG-TCA-AGG-AGG	23,358-31,627	8,269	221
	DV09	ACT-TTC-TGT-ACG-ACG-AGT-TCC			
5	DV10	TTG-CAT-GAG-TGT-CAA-GGG	31,406-39,609	8,203	255
	DV11	GAC-GTA-TCC-TCT-CTC-AAC-G			
6	DV12	GAG-GAC-GAT-GAC-TAC-AGC	39,354-47,443	8,089	146
	DV13	AGT-ACT-TTC-TGT-ACA-TGT-CCT-TG			
7	DV14	AGT-ACT-TTC-TGT-ACA-TGT-CCT-TG	47,297-55,433	8,136	99
	DV15	AGT-ACT-TTC-TGT-ACA-TGT-CCT-TG			
8	DV16	ATG-CAA-CAA-TGG-TTT-CGC	55,334-62,712	7,378	287
	DV17	GTA-ATC-TTT-CCC-AGT-CTC-GG			
9	DV18	CTA-TGG-TGA-TGT-TTA-CCT-TTG-CG	62,425-71,413	8,988	247
	DV19	TAC-CTC-TTG-CAG-ATG-TGC			
10	DV20	TCC-TAC-TGT-GCA-TCT-TTC-C	71,166-78,738	7,572	149
	DV21	CAA-AGA-AGC-ATG-CAA-GCG			
11	DV22	CCG-TTT-ACG-ATC-GTG-ATA-CC	78,589-87,307	8,718	160
	DV23	CTG-TAG-ACT-CCT-TTC-ACC-C			
12	DV24	CTG-TAG-ACT-CCT-TTC-ACC-C	87,147-93,423	6,276	159
	DV25	CCA-CAA-GAA-TGT-TTG-CAC-C			
13	DV26	TTG-TCT-GAA-AGA-AAG-TCT-CTA-GCG	93,254-99,862	6,598	125
	DV27	AAC-TCG-TCA-ATC-ATG-GTC-C			
14	DV28	AAC-TCG-TCA-ATC-ATG-GTC-C	99,737-105,901	6,164	278
	DV29	GCT-TTA-CTT-TCA-ATG-AAT-TCA-TCG-G			

Table S3. PCR conditions for amplification of FV3 fragments.

Cycles	Step	Temperature	Time
1	Initial Denaturation	95 °C	1 min
40	Denaturation	95 °C	15 s
	Annealing	57 °C	15 s
	Extension	72 °C	5 min
1	Final Extension	72 °C	10 min
1	Cooling	8 °C	∞

Table S4. qPCR primers and probes.

**qPCR primers and probes**					
**Target**	**Primer**	**Sequence (5'-3')**	**Position in FV3 (NC_005946.1) or LMBV (MK681856)**	**Product size (bp)**	**Dye/quencher**
FV3	Forward	ACG-CCA-CCA-CGT-ACT-TTG-TC	97,436-97,543	108	FAM/BHQ1
	Reverse	AAA-ACT-GCT-GCC-CGA-AAG-CC			
	Probe	CCA-AGC-TGC-CGT-CTC-TGG-CTG-CCA-A			
LMBV [65]	Forward	TGA-TTG-GCA-ACA-CTA-GCG-ATC-T	97,012-97,073	62	FAM/BHQ1
	Reverse	CCT-AGC-TCC-TGC-TTG-ATC-GG			
	Probe	TCA-ATC-CCG-CCC-CCG-CC			
